# Sex differences in the combined effect of chronic stress with impaired vascular endothelium functioning and the development of early atherosclerosis: The Cardiovascular Risk in Young Finns study

**DOI:** 10.1186/1471-2261-10-34

**Published:** 2010-07-12

**Authors:** Nadja Chumaeva, Mirka Hintsanen, Markus Juonala, Olli T Raitakari, Liisa Keltikangas-Järvinen

**Affiliations:** 1 Institute of Behavioral Sciences, University of Helsinki, P.O. Box 9, 00014 Helsinki, Finland; 2 Division of Medical Problems of Cell Biology, Institute of Cell Biophysics, Institutional Street 3, Pushchino, Moscow region 142290, Russia; 3 Research Centre of Applied and Preventive Cardiovascular Medicine, University of Turku, Kiinanmyllynkatu 10, 20520 Turku, Finland; 4 Department of Medicine, University of Turku and Turku University Hospital, P.O. Box 52, 20521 Turku, Finland; 5 Department of Clinical Physiology, University of Turku, P.O. Box 52, 20521 Turku, Finland

## Abstract

**Background:**

The syndrome of vital exhaustion (VE), characterized by fatigue and irritability, may contribute to an increased risk of atherosclerosis. The aim of the study was to explore sex differences in the interactions of VE with endothelial dysfunction and VE with reduced carotid elasticity, the important contributors to the development of early atherosclerosis, on preclinical atherosclerosis.

**Methods:**

The participants were 1002 women and 719 men aged 24-39 examined in the Cardiovascular Risk in Young Finns study. Vital exhaustion was measured using the Maastricht Questionnaire. Preclinical atherosclerosis was assessed by carotid intima-media thickness (IMT), endothelial function was measured by brachial flow-mediated dilatation (FMD), and arterial elasticity by carotid artery compliance (CAC) using ultrasound techniques.

**Results:**

We found a significant CAC x VE interaction for IMT only for the men. Our results imply that high VE level significantly related to high IMT levels among the men with low CAC, but not among the women with low CAC or among the women or men with high CAC. No significant FMD x VE interactions for IMT for the women or men were found.

**Conclusions:**

High VE may exert an effect on IMT for men with impaired arterial elasticity. The results suggest that high vitally exhausted men with reduced arterial elasticity are at increased risk of atherosclerosis in early life and imply men's decreased stress coping in relation to stressful psychological coronary risk factors.

## Background

Coronary heart disease (CHD) is the leading cause of morbidity and mortality in the industrialized world [1]. Atherosclerosis is the pathogenic process that underlies most cardiovascular diseases, including the pathology of CHD [2]. According to the prognosis of the World Health Organization, stressful life events and chronic psychosocial stress will be the most harmful risk factors for the development of cardiovascular diseases in the near future [3]. Recent studies have shown that chronic life stress is a significant risk factor for cardiovascular mortality [4], and chronic psychosocial stress has been found to contribute on endothelial dysfunction [5], fostering, therefore, the progression of atherosclerosis [6].

It is known that endothelial dysfunction is a marker of cardiovascular [7] and atherosclerotic [8] risk and it triggers the first step of atherosclerosis [9]. The alterations in the functioning of the vascular endothelium may influence the associations between the risk factors and atherosclerosis progression [10]. Brachial flow-mediated dilatation (FMD) serves as an adequate non-invasive measure of endothelial function [11] and carotid arterial compliance (CAC) as a valid index of arterial elasticity [12]. CAC may also serve as an additional indicator of early atherosclerosis [7,13]. Reduced FMD [14] and decreased arterial compliance [15] have been shown in the Cardiovascular Risk in Young Finns (CRYF) study to be related to the increased intima-media thickness (IMT), whereas increased IMT correlates with coronary atherosclerosis [16]. IMT is a marker of preclinical atherosclerosis, and it has been found to predict future cardiovascular disease independent of traditional risk factors [17].

Vital exhaustion (VE) has been considered to be a type of chronic mental stress, and it is characterized by heightened irritability, fatigue and demoralizing feelings [18]. Vital exhaustion has been associated with coronary events [18] and coronary heart disease [19]. A state of VE has shown to be one of the risk indicators for myocardial infarction [20] and for stroke [21] and it has been suggested to be predictive for atherothrombotic events [22,23]. Our recent studies have shown that VE may contribute to an increased risk of early atherosclerosis in young healthy adults [24,25].

Several years ago, Appels has described a state of VE, characterized by the symptoms of tiredness, physical exhaustion and feeling of hopelessness as a prodromal state in cardiovascular patients [18]. Appels has suggested that symptoms of VE can precede CHD events, perhaps, through the neuroendocrine mechanisms. Later, a number of additional linking mechanisms, such as proinflammatory and procoagulant changes and increased pathogen burden have been found to participate in VE symptomatology as well as in cardiovascular and chronic stress relationships [26-31]. The exploration of these mechanisms is highly important, because atherosclerosis has recently been shown to be a chronic inflammatory process [32]. However, neuroendocrine mechanisms cannot be excluded from the possible relations between VE and atherogenesis. The HPA axis [33] and the sympathetic-parasympathetic balance [34] as well as inflammatory mediators have been suggested to be involved in the psychological stress-related atherogenesis [35]. Moreover, atherogenic process has been considered to be a neurogenic phenomenon resulted from ANS imbalance towards a state of sympathetic hyperactivity [36]. In accordance with this hypothesis, the harmful influence of cardiovascular risk factors in relation to atherosclerosis may be mediated by the interactions between ANS regulation and endothelial function [37]. Chronic stress-related neuroendocrine mechanisms can play a role in the development of endothelial dysfunction [35]. Endothelial dysfunction may, in turn, lead to structural alterations of the arterial walls [38]. Based on these findings we hypothesized that a high level of VE in combination with impaired endothelial function or reduced arterial elasticity may be related to a disproportionately high development of preclinical atherosclerosis (increased IMTs).

Data from several studies demonstrate that cardiovascular mortality and the lifetime risk of the CHD development is higher in men compared to women, especially in stressful life conditions [1]. Men from industrialized eastern European countries demonstrated more VE and fewer effective coping strategies than women and men living in the West [39]. It has been shown that women develop atherosclerosis later than men probably because of the estrogen's protective role [40]. In our recent study, an increased risk of atherosclerosis has been shown in men compared to women [41]. Based on these findings we hypothesized that the effect of high VE in combination with impaired endothelial functioning in relation to the risk of atherosclerosis is most pronounced in men. More specifically, we expected that a high level of VE in combination with impaired endothelial function (low FMD) or reduced arterial elasticity (low CAC) would be related to increased risk of atherosclerosis (increased IMTs) in men. We studied the interaction of VE and endothelial dysfunction, measured by FMD, on preclinical atherosclerosis, assessed by carotid IMT, in young healthy men and women aged 24-39 years concentrating on sex differences. In addition, we investigated the interaction of VE and CAC on carotid IMT separately for the men and women. We also took into account the effects of cardiovascular risk factors associated with ultrasound variables in the CRYF study [10,14,15,42]: serum LDL-cholesterol levels, serum HDL-cholesterol levels, triglyceride levels, systolic and diastolic blood pressure.

## Methods

### Subjects

The participants are from the sample of the ongoing prospective epidemiological Cardiovascular Risk in Young Finns study [43] which is investigating the risk factors and precursors of cardiovascular diseases and their determinants in 3596 healthy children, adolescents and young adults from different parts of Finland [43]. In the CRYF sample, carotid IMT, CAC and brachial FMD were measured in 2001. Complete data on carotid and brachial artery ultrasound measurements were available for 2109 subjects aged 24 to 39 years. Vital exhaustion was also measured in 2001; valid VE questionnaires were obtained from 2080 participants. The final sample of the current study comprised 1002 women and 719 men.

The study followed the guiding principles of the Helsinki Declaration and was approved by the local ethics committees. All the subjects gave their written, informed consent.

### Vital exhaustion

Vital exhaustion was assessed with the Maastricht Questionnaire (MQ), a 21-item checklist of signs and symptoms of exhaustion [44]. The MQ has been designed for self-application. It has been specially developed to assess feelings of exhaustion. The MQ consists of 21 questions asking about symptoms of VE state: increased irritability, unusual fatigue, loss of energy and feelings of demoralization, all scored as absent or present. An analysis of the items which are included in MQ and indicate VE feelings has shown the significant associations between the items and cardiovascular diseases [44]. The questionnaire consisted of the following questions regarding the presence or absence of feelings of fatigue, irritability and demoralization: "Do you ever wake up with a feeling of exhaustion and fatigue? Do little things irritate you more lately then used do? Do you feel you want to give up trying? Do you lately feel more listless than before?" [44]. Each of the items was rated on a three-point scale, ranging from 0 to 2. The answer's variables were: "no" = 0, "I cannot say" = 1 and "yes" = 2. The mean score of all the items was used to index the level of VE. Cronbach's alpha was 0.92, indicating good reliability. The questionnaire was sent to the participants to be completed at home.

### Ultrasound imaging

Ultrasound studies of the carotid and brachial arteries were performed using Sequoia 512 ultrasound mainframes (Acuson, Mountain View, CA, USA) with 13.0 MHz linear array transducer, as previously described [14]. To assess intra-individual reproducibility of ultrasound measurements, 57 subjects were re-examined 3 months after the initial visit (2.5% random sample).

### Carotid intima-media thickness, IMT

Carotid IMT was measured on the posterior (far) wall of the left carotid artery. At least four measurements were taken approximately 10 mm proximal to the bifurcation to derive mean carotid IMT. The between-visit coefficient of variation (CV) of IMT measurements was 6.4% and the intra-observer CV was 3.4% [10].

### Brachial flow-mediated dilatation, FMD

To assess brachial FMD, the left brachial artery diameter was measured both at rest and during reactive hyperemia. Increased flow was induced by inflation of a pneumatic tourniquet placed around the forearm to a pressure of 250 mmHg for 4.5 min, followed by a release [14]. Arterial diameter was measured at end-diastole at a fixed distance from an anatomic marker at rest and 40, 60 and 80 s after cuff release. The vessel diameter in scans after reactive hyperemia was expressed as the percentage relative to the resting scan. The average of three measurements at each time point was used to derive the maximum FMD (the greatest value between 40 to 80 s). All ultrasound scans were analyzed by a single reader blinded to the subject's details [14]. The between-visit CV for brachial diameter was 3.2% and for FMD 26.0% [14]. Intra-observer CV was 1.2% for brachial diameter and 15.3% for FMD [10].

### Carotid artery compliance, CAC

Several moving image clips of the beginning of the carotid bifurcation and the common carotid artery with a duration of 5 s were recorded and stored in digital format for subsequent off-line analysis. The best quality cardiac cycle was selected from the clip images. The carotid diameter was measured at least twice (spatial measurements) in end-diastole and end-systole, respectively. The mean of the measurements was used as the end-diastolic or end-systolic diameter. Blood pressure was measured during the ultrasound study with an automated sphygmomanometer (Omron M4, Omron Matsusaka Co., Ltd, Japan). Ultrasound and concomitant brachial blood pressure measurements were used to calculate carotid compliance (CAC = [(Ds - Dd)/Dd]/(Ps - Pd), where Dd is diastolic diameter, Ds is systolic diameter, Ps is systolic blood pressure, and Pd is diastolic blood pressure). The between-visit CV was 2.7% for diastolic diameter and 16.3% for CAC [15]. Intra-observer CV was 1.4% for diastolic diameter and 13.6% for CAC [10].

### Clinical Characteristics and Cardiovascular Risk Factors

We took into account the effects of serum LDL-cholesterol levels, serum HDL-cholesterol levels, triglyceride levels, systolic and diastolic blood pressure. For the determination of serum lipoprotein levels, venous blood samples were drawn after an overnight fast. All measurements of lipid levels were performed in duplicate in the same laboratory. LDL-cholesterol concentration was calculated by the Friedewald formula [45]. Standardized enzymatic methods were used for measuring levels of triglycerides and HDL-cholesterol. Details of the methods have been reported elsewhere [46]. Blood pressure was measured with a random-zero sphygmomanometer. Average of three measurements was used in the analysis. Blood pressure was included into analyses because it had previously been shown to predict IMT among young adults from 33 to 39 years [42].

### Statistical analyses

The interactions between FMD and VE and between CAC and VE in predicting carotid IMT were tested using linear regression analyses (SPSS Version 16.0). The main effects of age, VE, FMD and baseline brachial diameter were included in the regression analyses when examining the interaction between FMD and VE in predicting IMT. The main effects of age, VE and CAC were included in the regression model testing the interaction between CAC and VE. Vital exhaustion and ultrasound measures included in the interactions were centralized. The main effects of the risk factors (serum LDL-cholesterol levels, serum HDL-cholesterol levels, triglyceride levels, systolic blood pressure and diastolic blood pressure) were additionally included into the FMD x VE interaction model as well as into the CAC x VE interaction model adjusted for the cardiovascular risk factors. The three-way interactions FMD x VE x sex and CAC x VE x sex were also tested. The analyses were carried out for the whole sample and among women and men separately.

## Results

The mean values for the study parameters are presented in Table 1.

**Table 1:** Characteristics of the study participants (Total N = 1721)

	*Women*			*Men*			
***Variable***	***Mean***	***(SD)***	***N***	***Mean***	***(SD)***	***N***	***p-value***

Age, years (24-39)	***31.58***	5.03	1002	***31.76***	5.08	719	*0.467*^*ns*^
Baseline brachial diameter, (mm)	***3.11***	0.32	949	***3.99***	0.44	653	0.000***
Flow-mediated dilatation, FMD (%)	***8.81***	4.55	949	***6.92***	4.04	653	0.000***
Carotid artery compliance, CAC (%/10 mmHg)	***2.31***	0.77	999	***2.01***	0.66	716	0.000***
Carotid intima-media thickness, IMT (mm)	***0.57***	0.08	1002	***0.59***	0.10	719	0.000***
Vital exhaustion, VE	***0.47***	0.39	1002	***0.35***	0.34	719	0.000***
LDL-cholesterol (mmol/l)	***3.17***	0.77	997	***3.44***	0.91	714	0.000***
HDL-cholesterol (mmol/l)	***1.40***	0.31	997	***1.17***	0.28	714	0.000***
Triclycerids (mmol/l)	***1.17***	0.58	997	***1.44***	0.77	714	0.000***
Systolic BP (mm Hg)	***117.30***	13.06	601	***116.36***	13.18	491	*0.236 *^*ns*^
Diastolic BP (mm Hg)	***71.29***	10.66	601	***70.37***	10.96	491	*0.161 *^*ns*^

*** < .001; ^ns ^- non-significant.

HDL = high density lipoprotein, LDL = low density lipoprotein, BP = blood pressure.

P-values refer to the mean differences between the men and women.

Linear regression analyses showed that FMD as well as CAC were not related to IMTs for the women or men (p = 0.391, p = 0.626 and p = 0.595, p = 0.124, respectively). VE was not related to IMT among the women (p = 0.744), whereas VE was positively related to IMT among the men (p = 0.036).

Table 2 presents the results of linear regression analyses of age and cardiovascular risk factors on carotid IMT among the women and men. Age was positively associated with IMT both in the women and men. HDL-cholesterol level was associated with IMT among the women.

**Table 2:** Regression analysis of age and cardiovascular risk factors on carotid IMT among the women (N = 596) and men (N = 486)

	β	t-value	p-value
**Women**			
Age	0.32	8.15	0.000***
LDL-cholesterol (mmol/l)	0.02	0.62	0.537
HDL-cholesterol (mmol/l)	-0.08	-1.97	0.049*
Triclycerids (mmol/l)	-0.02	-0.50	0.615
Systolic BP (mm Hg)	0.09	1.66	0.096
Diastolic BP (mm Hg)	0.04	0.70	0.485
**Men**			
Age	0.34	7.56	0.000***
LDL-cholesterol (mmol/l)	0.07	1.67	0.095
HDL-cholesterol (mmol/l)	-0.02	-0.48	0.630
Triclycerids (mmol/l)	-0.07	-1.46	0.144
Systolic BP (mm Hg)	0.01	0.14	0.889
Diastolic BP (mm Hg)	0.01	0.24	0.812

*< .05, ***< .001.

HDL = high density lipoprotein, LDL = low density lipoprotein, BP = blood pressure.

### The flow-mediated dilatation x vital exhaustion interaction in relation to intima-media thickness

Table 3 presents the results of linear regression analyses of the interactions of FMD with VE for IMT performed a) for all participants together, b) separately for the women and men.

**Table 3:** Regression analysis of the interaction between vital exhaustion and flow-mediated dilatation/carotid artery compliance in relation to IMT

	**R**^2^*	**R**^**2**^ change**	p	N
**FMD responses**				
FMD x VE^1^	0.129	0.000	0.984	1602
*(all FMD responses)*				
#FMD x VE^1^	0.175	0.000	0.678	985
*(all FMD responses)*				
FMD x VE^1 ^(women)	0.109	0.000	0.796	949
#FMD x VE^1 ^(women)	0.129	0.001	0.335	555
FMD x VE^1 ^(men)	0.136	0.001	0.483	653
#FMD x VE^1 ^(men)	0.189	0.000	0.631	430
**CAC responses**				
**CAC x VE^1^**	0.121	0.001	0.250	1715
*(all CAC responses)*				
**#CAC x VE^1^**	0.167	0.001	0.358	1076
*(all CAC responses)*				
CAC x VE^1 ^(women)	0.108	0.000	0.651	999
#CAC x VE^1 ^(women)	0.135	0.003	0.185	593
***CAC x VE***^***1 ***^***(men)***	***0.124***	***0.005***	***0.046***	***716***
***#CAC x VE***^***1 ***^***(men)***	***0.177***	***0.009***	***0.025***	***483***

*Note*. VE = vital exhaustion, FMD = flow-mediated dilatation (%), CAC = carotid artery compliance (%/10 mmHg).

^1 ^The main effects were included in each analysis, but they are not presented in the table.

*Calculated for the whole model.

**Refers to the interaction term.

#Additionally adjusted for cardiovascular risk factors: LDL-cholesterol, HDL-cholesterol, triglycerides, systolic and diastolic blood pressure.

The interaction of FMD and VE for IMT was found to be non-significant for the whole sample (p = 0.984) and when the women and men were analyzed separately (p = 0.796 and p = 0.483, respectively). The associations remained non-significant after adjustments for traditional cardiovascular risk factors. The three-way interaction analysis showed that the FMD x VE x sex interaction was non-significant (p = 0.138) even after adjusting for the cardiovascular risk factors (p = 0.070) (Table 4).

**Table 4:** The three-way interactions between sex, VE and FMD on carotid IMT and between sex, VE and CAC on carotid IMT

	**R**^2^*	**R**^**2**^ change**	p	N
**FMD responses**				
FMD x VE x sex^1^	0.130	0.001	0.138	1602
#FMD x VE x sex^1^	0.177	0.003	0.070	985
**CAC responses**				
***CAC x VE x sex***^***1***^	***0.113***	***0.004***	***0.005***	***1715***
***#CAC x VE x sex***_***1***_	***0.159***	***0.006***	***0.006***	***1076***

*Note*. VE = vital exhaustion, FMD = flow-mediated dilatation (%), CAC = carotid artery compliance (%/10 mmHg).

^1 ^The main effects were included in each analysis, but they are not presented in the table.

*Calculated for the whole model.

**Refers to the interaction term.

#Additionally adjusted for cardiovascular risk factors: LDL-cholesterol, HDL-cholesterol, triglycerides, systolic and diastolic blood pressure.

### The carotid artery compliance x vital exhaustion interaction in relation to intima-media thickness

Table 3 also shows the results of linear regression analyses of the interactions of CAC with VE for IMT a) for all participants together, b) separately for the women and men. The interaction of CAC and VE for IMT was non-significant when the analysis was carried out for the whole sample (p = 0.250) and for the women (p = 0.651). However, the CAC x VE interaction was significantly related to IMT in the men (p = 0.046, N = 716). This association remained significant after adjustments for cardiovascular risk factors only among the men (p = 0.025) (Table 3). The results of the three-way interaction analysis demonstrated a significant CAC x VE x sex interaction (p = 0.005). This interaction remained significant in a model adjusted with cardiovascular risk factors (p = 0.006) (Table 4).

Linear regression analyses conducted separately for the high (N = 281) and low (N = 435) CAC men (median split) showed that high VE significantly related to high IMT levels among the men with low CAC (β = 0.113, p = 0.019, N = 435), but not among the men with high CAC (β = 0.031, p = 0.609, N = 281).

## Discussion

The different roles of cardiovascular risk factors in men and women have recently been discussed [47]. Many risk factors, including stress-related psychosocial coronary risk factors (e. g., social isolation, VE) have been reported to have a greater impact on CHD progression in men compared to women, especially in stressful psychosocial conditions [1]. In line with these findings and with our hypothesis, we found a significant CAC x VE interaction for IMT only in the men. This association persisted after all adjustments, showing that age and traditional cardiovascular risk factors may have an additional harmful pressure in relation to atherosclerosis. The results of the three-way interactions analyses represent that the interaction between CAC and VE are statistically different between men and women. Our results imply that high VE significantly predicted high IMTs among the men with low CAC, but not among the women with low CAC or among the women or men with high CAC.

In the previous studies, we found the interactions of VE and acute stress reactivity/recovery with preclinical atherosclerosis only in the men [24], and a differential effect of VE in men compared to women [34]. In addition, chronic stress has been shown to be associated with IMT only in men in the recent CRYF study [48]. Furthermore, compared to women, men with high levels of cardiovascular risk factors have been found to demonstrate decreased elasticity and a significantly increased risk of structural atherosclerosis progression [41]. Taken together, our current results imply that the effects on atherosclerosis are more pronounced in high vitally exhausted men with decreased CAC compared to women having the same levels of VE and CAC. These findings are in line with the concept of the female sex hormone's (estrogen) protective role in connection with atherosclerosis progression [40]. Vital exhaustion belongs to one of the types of prolonged (chronic) stress. Chronic psychological stress has often been linked to estrogens in women. Thus, preventive role of estrogen on depression-like behavior has been found in the latest research when estrogen therapy has been applied on the animal depression model [49]. On the other hand, estrogens have been reported to decrease total cholesterol and LDL-cholesterol levels [50,51]. A vasodilatory effect of estrogens on the walls of vessels has been shown, and, in addition, the atheroprotective effect via inhibition of smooth-muscle cell proliferation [52]. Moreover, estrogens have been found to decrease arterial stiffness [53], therefore, estrogens may increase arterial compliance (high arterial compliance is an indicator of good arterial elasticity, whereas high arterial stiffness indicates impaired arterial elasticity). Our findings are also in accordance with the idea of men's decreased stress coping [1], as well as with the response-to-injury model of atherosclerosis development [9].

The possible mechanism explaining the interactions of VE and CAC on atherosclerosis progression could be participation of the HPA axis [33] and the sympathetic-parasympathetic balance [34] in stress-related vascular disease development, possibly by triggering endothelial dysfunction [35], which can be related to structural alterations of the arterial walls [38], increasing the sensitivity of the vasculature to the harmful influence of risk factors and contributing to the pathogenesis of the atherosclerotic process. In addition, proinflammatory [28-31] and procoagulant alterations [26,27] have been found to play a role in VE and atherogenesis relations. Van der Ven and coauthors [28] have found that VE is associated with (1) increased levels of cytokines, (2) increased procoagulant activity and (3) multiple herpes virus infections. In addition, elevated levels of both serum cytokines concentrations and tumor necrosis factor alpha have recently been reported to be associated with VE in patients with cardiovascular risk factors [54]. Increased cytokines levels have also been reported in patients, who are exhausted after percutaneous coronary intervention [31]. It has been shown earlier, that cytokines may affect the brain and evoke the sense of life discomfort and feelings of fatigue and tiredness [55-57]. On the other hand, the relationship between endothelial dysfunction and inflammatory processes in the walls of vessels, which can foster atherosclerosis development, have recently been reported in the CRYF study [58]. Endothelial dysfunction has been considered to be an earlier indicator for the structural changes of the arterial walls [38]. Reduced arterial compliance reflects structural abnormalities/alterations of the walls of vessels, which are associated with the diseases and/or age [38]. In addition, the associations between VE and body mass index, suggested that reducing VE levels can play a role in reducing the prevalence of obesity have recently been found in ARIC study [59]. On the other hand, it has been demonstrated in the CRYF study, that young adults (aged 24-39 years) with metabolic syndrome have increased carotid IMTs and decreased CAC [60]. Possible links between obesity and VE [59], between metabolic factors and carotid atherosclerosis, which has been shown earlier [61], as well as between the metabolic syndrome and CAC [60] offer another potential explanation for the relations found between VE, CAC and carotid atherosclerosis.

No significant FMD x VE interactions for IMT in the women or men were found. These results are in agreement with a recent findings that CAC is more closely related to coronary risk than FMD [62] and consistent with the idea that in some conditions arterial compliance/arterial stiffness may be a more sensitive and more effective risk marker than FMD [7,62], because many risk factors can influence the elastic properties of vessels [7,12,63]. Indexes of arterial elasticity have been found to be highly important for identification of cardiovascular events risk and for determining the level of intervention [7]. Thus, the predictive value of aortic stiffness on primary stroke in hypertensive patients has been shown [64]. Future studies are needed to confirm the predictive values of arterial stiffness on cardiovascular events. In addition, patients at risk for cardiovascular events may benefit from earlier recognition of impaired arterial compliance and vascular abnormality. Earlier recognition and a reduction of arterial stiffening may decrease a risk and prevent cardiovascular events [38]. Several non-invasive arterial elasticity tests have recently been created by various medical-research companies and some tests, such as measuring of arterial stiffness and arterial compliance, are under clinical evaluation.

Vital exhaustion was shown in the present study to exert an effect on IMT in the young men with impaired arterial elasticity. The distinction of VE from depression remains a subject of discussion. Exhaustion symptoms such as sleep alterations and feelings of weakness overlap with depressive symptomatology. It has been found in the comparative depression/vital exhaustion study that VE and depressive symptomatology correlated strongly (shared a common variance of 38%: [65]). However, a "depressed" mood, the key symptom of depression, is usually absent in exhausted individuals [66]. Many studies have distinguished VE from depression, in that exhausted subjects have characterized by the absence of the main symptoms for depressive disorders: feelings of guilt, sadness, or feelings of worthlessness. Vitally exhausted subjects are typically characterized by demoralization, whereas the lowered self-esteem is a symptom of depression [19]. In the study, which assesses the differences between depression and VE in 12640 participants from Hungary, most depressed subjects (77%: [65]) investigated have been found to be exhausted, but cognitive and mood disturbances, the important symptoms of depression, have been shown to be usually absent in exhausted subjects [65]. In line with these findings and with [66] the depressive symptoms have been demonstrated to be a distinct from the concept of VE in 822 participants studied in Augsburg Cohort Study [67]. In addition, depressive symptoms and VE have been presented to be differentially related to behavioral risk factors for cardiovascular diseases: VE has been associated with increased probability of cardiovascular disorders and history of cardiovascular treatment, whereas depressive symptomatology with increased risk for illegal drug uses, alcohol abuse, congenital disorders, disabilities and hostility [65]. Finally, depression has typically considered to be a disease, whereas VE - a psychological state.

### Methodological considerations

There are some limitations in the present study. First, we measured carotid IMT from the left carotid artery, not from the internal carotid artery. However, previous data support the use of the common carotid artery IMT in both studies of risk factor associations and cohort studies [68,69]; the reproducibility of our measurements is comparable with other reports [69].

Second, we found relatively large within-subject long-term variation in FMD [14] and CAC measurements [15], which is in agreement with previous reports [70-72]; the long-term reproducibility of the carotid and brachial diameter measurements was very good, which suggests that much of the variation in CAC and FMD is due to physiological fluctuation. However, the large variability of FMD and CAC is a limitation.

The present analysis was conducted in participants aged 24 to 39. Our results cannot be generalized to older individuals with more definite atherosclerosis. Also, owing to the cross-sectional nature of the present study, it is impossible to make statements regarding atherosclerosis progress. We conducted several analyses, but not as many significant associations were found as was expected. Therefore, the possibility of a chance finding cannot be excluded. The CAC and VE interaction explains 0.5% of the variance in IMT in the men. This is a small amount, and we need to mention this point as an additional limitation of the study. Taking these limits into consideration, our results need to be viewed cautiously and replicated in future research.

The strength of our study is a comparatively large population-based sample. Furthermore, we were able to examine both men and women, which is important as it has been repeatedly shown that results related to cardiovascular diseases cannot be generalized from men to women or vice versa. Our study focused on young adults and brought important information on atherosclerosis development in a stage when clinical symptoms of carotid artery disease are rarely seen.

## Conclusions

We can conclude that unlike women, young asymptomatic men having high VE in combination with reduced arterial elasticity are at increased risk of atherosclerotic progression in early life. This is in line with the findings reporting that many cardiovascular risk factors have a greater impact on CHD in men than in women, and in line with the concept of men's decreased stress coping in relation to stressful psychological conditions.

## Abbreviations

ANS: autonomic nervous system; BP: blood pressure; CAC: carotid artery compliance; CHD: coronary heart disease; CRYF study: Cardiovascular Risk in Young Finns study; CV: coefficient of variation; FMD: flow-mediated dilatation; HDL: high density lipoprotein; HPA: hypothalamic-pituitary-adrenocortical; IMT: intima-media thickness; LDL: low density lipoprotein; MQ: Maastricht Questionnaire; SD: standard deviation; VE: vital exhaustion.

## Competing interests

The authors declare that they have no competing interests.

## Authors' contributions

LK-J and OTR were responsible for planning the study and participated in the study design and coordination. NC was responsible for the data analysis and statistics and made substantial contribution to the conception and design. MH helped with data analysis and statistics. NC and MH had the main responsibility of the manuscript writing. LK-J, OTR and MJ have made substantial contribution to collecting and acquisition of data. All authors contributed in interpretation of data. Each author participated in drafting the manuscript and revising it. All authors have read and given final approval of the version to be published.

## Pre-publication history

The pre-publication history for this paper can be accessed here:

http://www.biomedcentral.com/1471-2261/10/34/prepub
